# Physical and chemical stability of expired fixed dose combination artemether-lumefantrine in uncontrolled tropical conditions

**DOI:** 10.1186/1475-2875-8-33

**Published:** 2009-02-25

**Authors:** Roger Bate, Richard Tren, Kimberly Hess, Amir Attaran

**Affiliations:** 1 Africa Fighting Malaria, Washington, D.C, USA; 2 American Enterprise Institute, Washington, D.C, USA; 3 Institute of Population Health & Faculties of Law and Medicine, University of Ottawa, Ottawa, Canada

## Abstract

**Background:**

New artemisinin combination therapies pose difficulties of implementation in developing and tropical settings because they have a short shelf-life (two years) relative to the medicines they replace. This limits the reliability and cost of treatment, and the acceptability of this treatment to health care workers. A multi-pronged investigation was made into the chemical and physical stability of fixed dose combination artemether-lumefantrine (FDC-ALU) stored under heterogeneous, uncontrolled African conditions, to probe if a shelf-life extension might be possible.

**Methods:**

Seventy samples of expired FDC-ALU were collected from private pharmacies and malaria researchers in seven African countries. The samples were subjected to thin-layer chromatography (TLC), disintegration testing, and near infrared Raman spectrometry for ascertainment of active ingredients, tablet integrity, and chemical degradation of the tablet formulation including both active ingredients and excipients.

**Results:**

Seventy samples of FDC-ALU were tested in July 2008, between one and 58 months post-expiry. 68 of 70 (97%) samples passed TLC, disintegration and Raman spectrometry testing, including eight samples that were post-expiry by 20 months or longer. A weak linear association (R^2 ^= 0.33) was observed between the age of samples and their state of degradation relative to brand-identical samples on Raman spectrometry. Sixty-eight samples were retested in February 2009 using Raman spectrometry, between eight and 65 months post-expiry. 66 of 68 (97%) samples passed Raman spectrometry retesting. An unexpected observation about African drug logistics was made in three batches of FDC-ALU, which had been sold into the public sector at concessional pricing in accordance with a World Health Organization (WHO) agreement, and which were illegally diverted to the private sector where they were sold for profit.

**Conclusion:**

The data indicate that FDC-ALU is chemically and physically stable well beyond its stated shelf-life in uncontrolled, tropical conditions. While these data are not themselves sufficient, it is strongly suggested that a re-evaluation of the two-year shelf-life by drug regulatory authorities is warranted.

## Background

Improving access to effective anti-malarial treatment is a priority of most donors, United Nations agencies and national governments. In Africa and Asia, where the disease is most prevalent, limited health care infrastructure complicates efforts to predict clinical demand, procure, distribute, and administer medicines during their approved shelf-life. This typically results in purchasers procuring too little medicine, causing stock-outs and excess mortality, or purchasing too much medicine, causing overstock and drugs expiring on the shelf [1].

This logistical tight-rope act is more complicated than ever because chloroquine, with a shelf-life of up to five years, has been superseded as the standard of care by artemisinin-based combination therapy (ACT), such as a fixed dose combination artemether-lumefantrine (FDC-ALU), with a shelf-life of only two years. The reduction in shelf-life has contributed to serious disruptions in treatment availability in clinics; reluctance among health care workers to adopt the higher standard of care; and practical questions about whether large-scale home treatment schemes for malaria are feasible [2-4]. Globally, the shorter shelf-life also reduces the margin for error that pharmaceutical manufacturers and large purchasers, like health ministries, have to move in lockstep and balance demand with supply – a balance that, when upset, has caused global shortages and price spikes [5].

Currently, FDC-ALU is one of the dominant forms of ACT. That it has a two-year shelf-life is a legacy of history. FDC-ALU was first registered with the drug regulatory authorities in Switzerland where, in accordance with International Conference on Harmonization guidelines, the shelf-life of a medicine is extrapolated from accelerated laboratory stability tests, usually by adding 12 months to the duration of those tests (e.g. if the accelerated laboratory stability tests are good at 12 months, the regulator can grant an approved shelf-life of 24 months) [6]. Approvals for over two years usually are not based solely on accelerated stability testing, but also on long-term stability data collected in real time (e.g. approval for a five-year shelf-life may require five years of tests). Other agencies, including the WHO, ordinarily do not question the two-year shelf-life that FDC-ALU has today.

The goal of this study was to assess the physical and chemical stability of expired FDC-ALU under tropical field conditions, as a preliminary step toward possibly reexamining and extending the authorized shelf-life. If future and more detailed studies indicate this step were warranted, one could potentially simplify the logistics and reduce the cost of ACT delivery, further displacing obsolete medicines, such as chloroquine, with implicit gains to treatment effectiveness.

## Methods

Seventy samples of expired FDC-ALU were collected at random from private pharmacies and kiosks in major cities of Kenya, Mozambique, Nigeria, Rwanda, Tanzania, Uganda and Zambia, as well as from malaria researchers' personal supplies. These samples had not been stored under ideal environmentally controlled conditions, but rather under heterogeneous (and often suboptimal) "field" conditions. To exclude flagrant counterfeits, packaging was visually inspected for correctness and the batch numbers were confirmed with the apparent manufacturer (no such counterfeits were found).

Sixty-eight of the samples were confirmed to have been manufactured by Novartis Pharmaceuticals AG (Coartem^® ^and Riamet^®^, which are identical products), and two samples were confirmed to have been manufactured by Cipla Ltd (Lumartem^®^). Unexpired manufacturers' products for use as analytical standards were procured either by purchase and donation (in the case of Coartem/Riamet) or by purchase alone (in the case of Lumartem). Further, to independently corroborate the active ingredient in the manufacturers' standards, a master FDC-ALU standard was obtained from the Global Pharma Health Fund (Frankfurt).

In July 2008, primary screening of samples was conducted by the Global Pharma Health Fund e.V. Minilab^® ^protocol, published elsewhere [7]. Semi-quantitative thin-layer chromatography (TLC) and disintegration tests were performed on each sample to determine tablet integrity and the presence and amount of active ingredients, relative to the master standard. Two tablets from each sample were separately tested, and per the Minilab^® ^protocol, a "pass" was awarded if 80% or more of both labeled active ingredients was present. (In the results no samples failed TLC, so there was perfect agreement between the duplicates.) Quality control of the Minilab was performed daily prior to field drug testing and consisted of performing TLC on Minilab-reference samples for the drugs being analyzed. In addition, Minilab reagents were quality control tested using reference samples when a new lot was introduced.

Secondary screening of samples was conducted using a portable Raman spectrometer, operated per the manufacturer's protocol (TruScan; Ahura Scientific, Inc., Wilmington, MA). This technique has been used in earlier studies to discern counterfeit from legitimate anti-malarials *in situ *[8,9]. Because Raman spectrometry is sensitive to the excipients in the tablet and not solely the active ingredients, samples of Coartem/Riamet and Lumartem must be tested with reference to the manufacturers' standards. The TruScan device's laser is capable of reading through blister packaging, but in these tests tablets were removed and held in a constant distance and orientation during testing. Samples were tested by Raman spectrometry on two occasions: initial testing at the same time as TLC (in July 2008), and retesting seven months later after being stored for that interval in a temperature-controlled office (in February 2009). Two tablets from each sample had their spectra measured using near infrared excitation (at 785 nm) and continuous readout of Raman shift spectrum from 250 to 2875 cm^-1^. The discrepancy between the spectra of the sample tablet and the standard is reflected in an overall figure of merit (p-value) generated by the TruScan device. It is expected that newer samples will have smaller discrepancies and higher p-values than older samples, which experience cumulative chemical degradation over time. A p-value below 0.05 corresponds to failure of the test.

## Results

The samples collected in seven African countries included FDC-ALU that ranged from one to 58 months past expiry date, with a mode and median age of two months at initial testing in July 2008 and nine months at retesting in February 2009.

Of the two Lumartem samples tested in July 2008, both passed TLC, disintegration and Raman spectrometry testing. Of the 68 Coartem/Riamet samples tested in July 2008, all passed thin-layer chromatography (TLC) testing, and nearly all passed disintegration and Raman spectrometry testing, with the following exceptions: both replicates of a Coartem/Riamet sample at 14 months past expiry date failed Raman spectrometry testing, and one replicate of a Coartem/Riamet sample 58 months past expiry date failed the disintegration test (see Table 1). The blister packages of the failing medicines showed visible signs of minor damage, which may have been sufficient to spoil the chemical and physical integrity of those tablets.

**Table 1:** Testing results for Coartem/Riamet by expiry date for Raman spectrometry, TLC and disintegration

		**July 2008 Test Results^a^**				**February 2009 Test Results^a^**	
		
**Expiry Date**	**Number of Samples**	**Number of Months Past Expiry Date**	**TLC**	**Disintegration**	**Raman**	**Number of Months Past Expiry Date**	**Raman**
June 2008	7	1	7/7	7/7	7/7	8	7/7

May 2008	39	2	39/39	39/39	39/39	9	39/39

February 2008	4	5	4/4	4/4	4/4	12	4/4

August 2007	7	11	7/7	7/7	7/7	18	7/7

May 2007	2	14	2/2	2/2	1/2	21	1/2

November 2006	3	20	3/3	3/3	3/3	27	3/3

October 2005	5	33	5/5	5/5	5/5	40	5/5

September 2003	1	58	1/1	0/1	1/1	65	0/1

**Total**			**68/68**	**67/68**	**67/68**		**66/68**

a. Results are total number of samples that passed testing/total samples tested.

**Figure 1:**
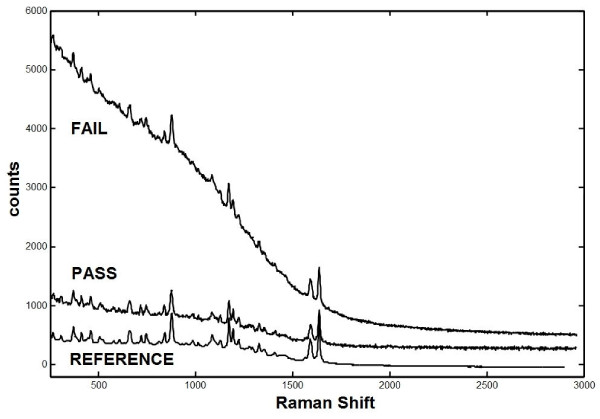
**Raman spectrum of passed and failed Coartem/Riamet and reference**. Plot of passed (p-value 0.388) and failed (p-value 0.004) Coartem/Riamet sample spectrum and reference spectrum, illustrated as counts (absolute measure of Raman intensity) versus Raman shift. The steep slope of the failed Coartem/Riamet sample is due to increased fluorescence of degradation products.

**Figure 2:**
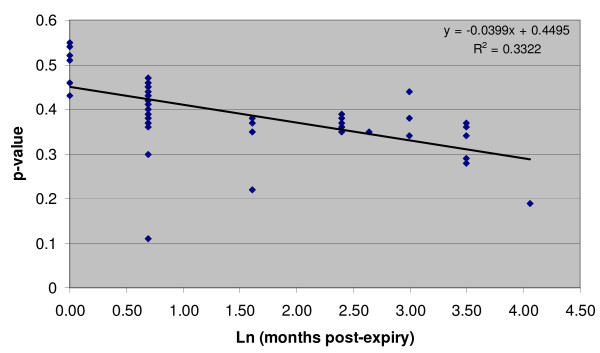
**Degradation of Coartem/Riamet over time**. Scatter plot of TruScan calculated p-values for all passing Coartem/Riamet samples versus natural log of months past expiry date.

Notwithstanding equivalent active ingredients, there were discernable differences between Coartem/Riamet and Lumartem samples in Raman spectrometry owing to differences in excipients. Samples of one manufacturer's medicine run against the other manufacturer's standard consistently failed the test (p < 0.05), both indicating that p-values for the two medicines are not comparable, and validating the Raman method as possessing sufficient sensitivity to "fail" a medicine for differences apart from the presence of active pharmaceutical ingredients. Within the Coartem/Riamet sample pool, Raman spectrometry demonstrated quality variations that were undetected by TLC. Figure 1 demonstrates example spectra for Coartem/Riamet samples that failed (p = 0.004) and passed (p = 0.388). For passing samples of Coartem/Riamet on initial testing, there appears to be a weak inverse relationship between sample p-value and the natural log transformed age of the product past date (Figure 2; R^2 ^= 0.33 by linear regression). This is consistent with the expected behavior of cumulative chemical degradation in samples over time, where the sample population reflects storage under heterogeneous field conditions and therefore varying rates of degradation.

**Figure 3:**
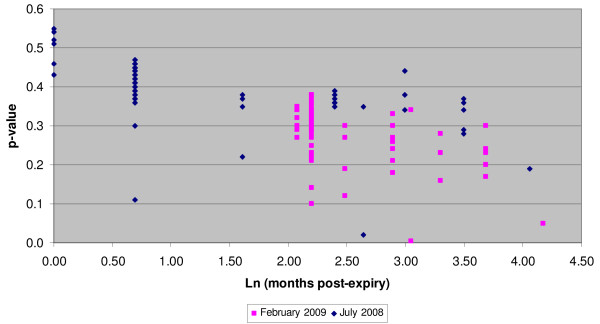
**Comparison of initial and retest Coartem/Riamet p-values**. Scatter plot of Coartem/Riamet initial and retest TruScan calculated p-values versus natural log of months past expiry date.

Of the 68 Coartem/Riamet samples retested in February 2009 using Raman spectrometry, the results were consistent with those from July 2008 (see Table 1) with the following exception: one replicate of the Coartem/Riamet sample that had passed at 58 months past expiry date in July 2008, failed at 65 months past expiry date and was therefore recorded as a borderline fail. Overall, 66 of 68 (97%) samples passed Raman spectrometry retesting. There were not sufficient tablets from every sample to retest TLC and disintegration. Over the seven month period from July 2008 to February 2009, the samples remained relatively stable despite a decline in the mean sample p-value from 0.39 to 0.28 respectively. This reduction in p-value is expected given the chemical degradation in samples over time. Figure 3 is a scatter plot of the sample p-values from initial testing and retesting.

Further, in a fortuitous observation, the authors found evidence that health ministry supply chains in some African countries are corrupted. In the course of matching batch numbers to verify the authenticity of the test samples, it was accidentally discovered that a batch of Coartem sold to the Nigerian Government strictly for public sector use, at concessional pricing had been illicitly diverted, and was on sale in a private pharmacy in Kenya. In another instance, two batches of Coartem sold to eight different African governments were found on sale in a private pharmacy in Rwanda.

## Discussion and conclusion

These results provide a strong indication FDC-ALU is a far more robust medicine in uncontrolled African conditions (*i.e. *tropical conditions) than is currently credited. Of 70 samples, the authors found that all of them contained an acceptable amount of active ingredients by the TLC method. Only one sample failed by the more exacting method of Raman spectrometry on initial testing, and another one sample failed disintegration testing. Overall on initial testing, 68/70 (97%) samples passed, including samples that were up to 33 months past their expiry date.

A limitation of this study is that its findings are generally only valid for Coartem/Riamet. The limitation is as expected: Coartem/Riamet was the first FDC-ALU to have entered the marketplace, and owing to a not-for-profit distribution agreement between Novartis AG and the WHO, it remains dominant. Few generic manufacturers, such as GVS Labs, Ajanta Pharma Ltd. and Jiangsu Yixing Forward Pharmaceutical Factory were manufacturing large quantities of FDC-ALU prior to 2006, and as such, their products are less available in the marketplace, and when they are encountered, are usually too recent to have passed their expiry date. The authors located two expired samples of Lumartem, manufactured by Cipla Ltd, which passed the battery of initial tests.

These data are useful, but not sufficient for a shelf-life extension. A more complete protocol would entail high performance liquid chromatography (HPLC) and/or tandem mass spectrometry analysis of degradation products; testing of package stability; testing for biological contamination; and possibly other tests as would be mandated by a drug regulator. However, while these studies are often done on medicines that have aged in controlled laboratory conditions, the advantage of this study is that it examines medicines kept in uncontrolled real-world conditions in tropical Africa. Since these conditions cannot always be accurately emulated in the laboratory – for example, a test designed to mimic stability of a product in a pharmacy's enclosed shelf may not approximate an open-air market pharmacy's presentation in full sunlight – these data should be viewed as complementary to the future laboratory studies previously recommended.

An advantage of this study is that it demonstrates reliable drug quality testing can be done without advanced analytical laboratories. The TLC and Raman techniques utilized in this study are not a substitute for the laboratory-based "gold standard" of HPLC or mass spectrometry, but are validated as "silver standard", which is easily field-deployable and which reliably detects deviations from a known reference medicine – and unlike an HPLC laboratory, the TruScan used in this study runs on battery power and fits in a person's hands. Drug quality surveillance from manufacturer to point-of-use likely requires both the gold and silver standard; it is not one or the other.

Until now, it has been assumed that supply-and-demand management for ACT could be simplified by stock-piling artemisinin or its derivatives, rather than finished products, as these tend to be over 99% stable when stored at 25 degrees Centigrade and 60% relative humidity [5]. While that is an attractive option, it also may not be necessary, as the stability of FDC-ALU is reasonably stable for about this same duration. The authors encountered apparently stable samples of 57 months in age on initial testing (= 24 months shelf-life + 33 months post-expiry).

A problem that all manufacturers of FDC-ALU have is that a 24-month shelf-life requires extraordinarily tight and difficult control over production schedules and supply chains. At the front end, the lead time is 14 months to cultivate and extract *Artemesia annua *and to carry out the manufacturing process; and at the back end, purchasers' demand 18 or more months of remaining product life upon receipt of product [10]. The former fact means that unless the manufacturer holds some product in inventory, then a purchaser who experiences a sudden rise in demand (as during an epidemic or complex emergency) cannot have that need met. The latter fact means that if the manufacturer actually holds product in inventory, it runs a serious financial risk because at only six months of age the product becomes unsalable. This is an invidious situation for manufacturers of FDC-ALU, who must choose between erring on the side of over-production and possible financial loss, or under-production and possible health crises. Such a tight market is also prone to price excursions, as have occurred for artemisinin before [5].

The unexpected observation of product diversion suggests that there is also scope to improve public sector medicine logistics in Africa. Such instances of diversion, besides being illegal, suggest that the public sector controls over FDC-ALU stocks are inaccurate. Inaccurate controls in this instance meant theft, but are also likely to be associated with mismanagement of medicines with a short shelf-life, leading to wastage.

The authors' findings argue for a reevaluation of the shelf-life of FDC-ALU, if confirmed by future studies using more advanced analytical techniques, which for the policy reasons already described would be very desirable. Improving access to medicines will require many different interventions that will vary from country to country. However, extending the shelf-life of ACTs would be of benefit in every country, which is why it should be a high priority, with hopefully many more lives saved as a result.

## Competing interests

RB received a travel grant within the past four years from Novartis AG. The other authors declare that they have no competing interests

## Authors' contributions

RB carried out all chemical analyses and prepared the first draft of the manuscript. RT and KH performed data analysis and edited the manuscript. AA performed literature review, statistical consultation and writing of the second draft of the manuscript. All authors read and approved the final manuscript.
